# Combing signal processing methods with algorithm priori information to produce synergetic improvements on continuous imaging of brain electrical impedance tomography

**DOI:** 10.1038/s41598-018-28284-2

**Published:** 2018-07-04

**Authors:** Haoting Li, Rongqing Chen, Canhua Xu, Benyuan Liu, Xiuzhen Dong, Feng Fu

**Affiliations:** Faculty of Biomedical Engineering, Fourth Military Medical University, 169 West Changle Road, Xi’an, 710032 China

## Abstract

Dynamic electrical impedance tomography (EIT) promises to be a valuable technique for monitoring the development of brain injury. But in practical long-term monitoring, noise and interferences may cause insufficient image quality. To help unveil intracranial conductivity changes, signal processing methods were introduced to improve EIT data quality and algorithms were optimized to be more robust. However, gains for EIT image reconstruction can be significantly increased if we combine the two techniques properly. The basic idea is to apply the priori information in algorithm to help de-noise EIT data and use signal processing to optimize algorithm. First, we process EIT data with principal component analysis (PCA) and reconstruct an initial CT-EIT image. Then, as the priori that changes in scalp and skull domains are unwanted, we eliminate their corresponding boundary voltages from data sets. After the two-step denoising process, we finally re-select a local optimal regularization parameter and accomplish the reconstruction. To evaluate performances of the signal processing-priori information based reconstruction (SPR) method, we conducted simulation and *in-vivo* experiments. The results showed SPR could improve brain EIT image quality and recover the intracranial perturbations from certain bad measurements, while for some measurement data the generic reconstruction method failed.

## Introduction

Dynamic electrical impedance tomography (EIT) is an imaging technique mostly used to recover the conductivity distribution changes inside a body^1^. In EIT, multiple electrodes are attached to the surface of the body. Then safe currents are applied across a particular pair of electrodes and boundary voltages are recorded on other electrodes. By switching the injection pair and repeating the process, data sets can be produced serially. Based on the data sets, we can finally reconstruct the tomographic images using a reconstruction algorithm. Being fast, non-invasive and low cost, EIT is high valued in the field of biomedical application^2,3^. For many brain diseases, lesions can cause cerebral conductivity changes^4^. So EIT has great potential for monitoring the development of brain injury or evaluating the efficacy of some relative treatments. In the previous studies, we validated EIT’s potential to unveil the development of stroke^5^, measured the intracranial conductivity changes in twist-drill drainage operation^6^ and monitored cerebral edema during dehydration treatment^7^.

However in the practical application, we found noises and interferences may impose severe adverse effects on brain EIT measurements and image quality. To our knowledge, random noise in measurements may cause large smearing artifacts and make image interpretation difficult^8,9^. And interferences like movement of patients, sweat, and volatilization of gel can reduce the electrode-skin contact status, severely deteriorating the signal quality^10,11^. As the time scale of brain injury monitoring is roughly hours, it is difficult for us to avoid these factors and maintain the measurements stable all the time.

For this challenging problem, introducing effective signal processing methods or optimizing reconstruction algorithm with priori information are helpful. Although most published works only used cerebral priori information to reduce modeling error^12,13^, we can easily apply it to the reconstruction following some examples of modified lung EIT algorithms^14,15^. And in the brain injury monitoring, we are accessible to patients’ CT or MRI, so it is convenient for us to obtain the cerebral priori information. More importantly, we think the two techniques can complete each other and produce synergetic improvements with proper combination. For most signal processing methods, they denoise data according to its amplitude or frequency characteristics. So priori information like structural information can provide a supplementary criterion for judging and eliminating noise and interferences. And for EIT algorithms, the vital regularization parameter, which influences reconstruction accuracy, is related to data quality^16,17^. So signal processing methods can be used to refine data sets and thus we can select a better regularization parameter to improve image quality.

**Figure 1:**
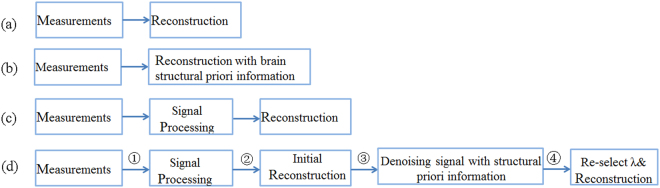
Flow charts of the generic reconstruction (GR) (**a**), priori information based reconstruction (PR) (**b**), signal processing based reconstruction (SR) (**c**) and signal processing-priori information based reconstruction process (SPR) (**d**).

Based on the above analysis, we made an optimization on brain EIT reconstruction process and proposed a signal processing-priori information based reconstruction (SPR) method. Figure 1 shows the flow chart of the generic reconstruction (GR) method, priori information based reconstruction (PR), signal processing based reconstruction (SR), and the SPR method. Different from the GR strategy which directly maps the measurements to EIT images, the SPR consists of four steps. Firstly, EIT measurements are processed by the principal component analysis (PCA) method. Then, with the processed data, we reconstruct an initial CT-EIT image using the damped least square algorithm^18^. Next, based on the structural priori information, we eliminate the boundary voltages induced by unwanted conductivity changes in the background area (skull and scalp domain). After the two-step denoising process, the EIT data sets only incorporate the changes of parenchyma. So we can finally re-select a local optimal regularization parameter and obtain better reconstructions.

To investigate the performance of SPR in brain EIT reconstruction, we conducted simulation and human experiments, in which typical errors like Gaussian noise and bad electrode-skin contact were considered. The results demonstrated that the SPR method was more efficient for improving continuous imaging of brain EIT.

## Results

### Simulation experiments

**Figure 2:**
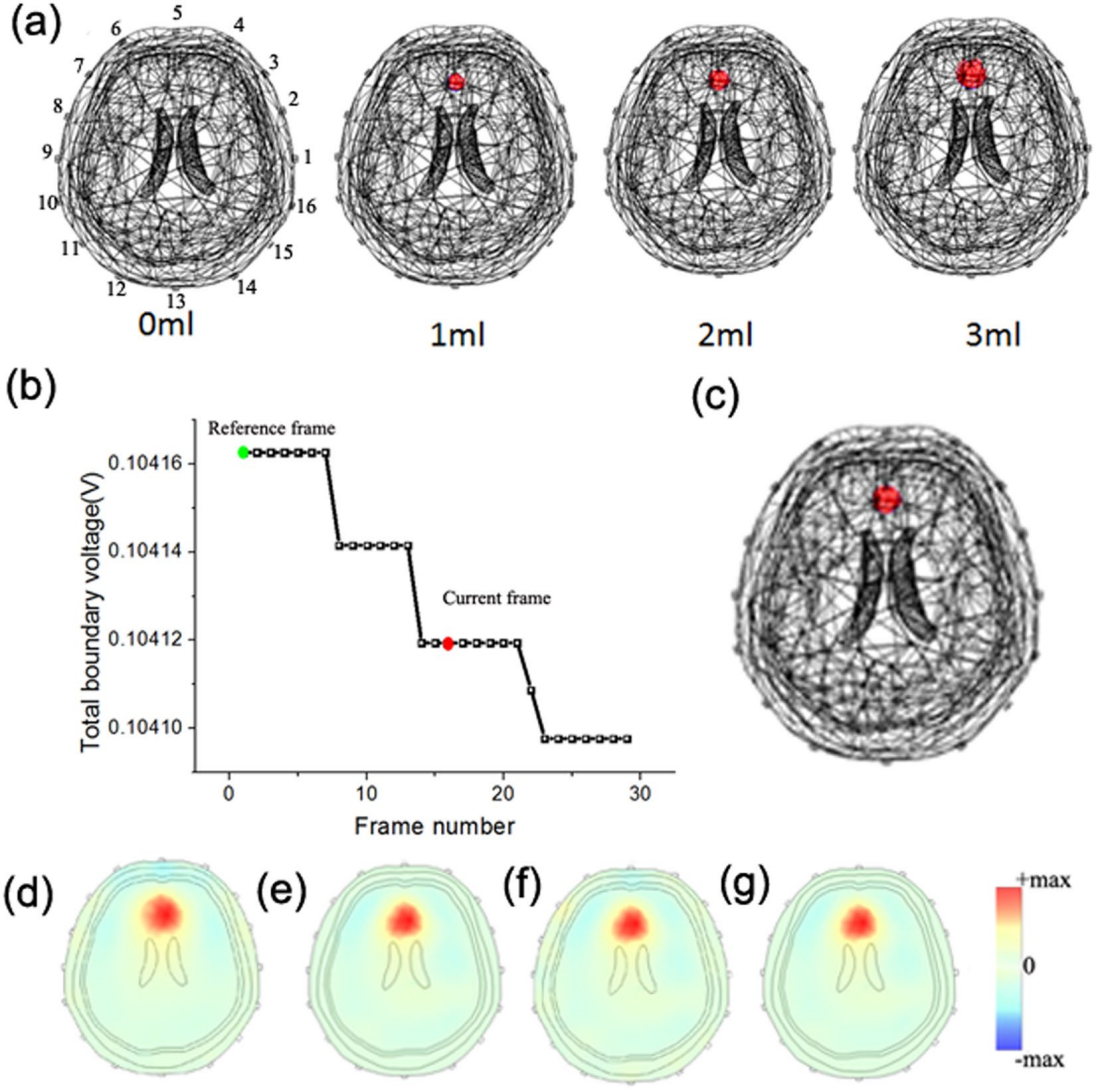
Simulated scenarios of monitoring cerebral hemorrhage with EIT: (**a**) the simulation of cerebral hemorrhage growth, (**b**) synthetic EIT measurements. The green and red points are respectively the reference frame and current frame, (**c**) True perturbation, (**d**–**g**) reconstruction results of GR, PR, SR and SPR without noise.

With a 3D FE head model, we simulated the process of detecting cerebral hemorrhage using EIT. And the detailed information of the 3D FE head model is presented in the method part. Figure 2(a) shows the simulated scenarios of cerebral hemorrhage growth. The red spheres represent perturbations and their conductivity values are set at 0.7 S/m, which is equivalent to the blood. By changing their volumes from 0 ml to 3 ml, we generated the corresponding boundary voltages. Each boundary voltage was duplicated for many times, and then we stitched and plotted them all together in sequence. As shown in Fig. 2(b), a curve, which could depict the time varying conductivity changes of cerebral hemorrhage growth, was synthesized. The curve’s horizontal axis shows the frame range and the vertical axis represents the total boundary voltage (the mean of voltage at all sensing electrodes). To convert the boundary measurements to difference voltages, we respectively set the reference and current frame at 0 ml and 2 ml. Figure 2(c) shows the true perturbation at the current frame. In Fig. 2(d–g), we reconstructed EIT images by GR (d), PR (e), SR (f) and SPR (g) without noise. In the ideal simulation experiments, these methods all got qualified images.

### Simulation results with Gaussian noise

To investigate whether SPR can make contributions to unveil intracranial conductivity changes from noisy measurements, we first add Gaussian noise to EIT data sets and compare its performances with other reconstruction processes. The MATLAB code for adding Gaussian noise is: y = awgn(x, SNR), where x is the difference data sets and SNR is the Signal-to-Noise Ratio.

**Figure 3:**
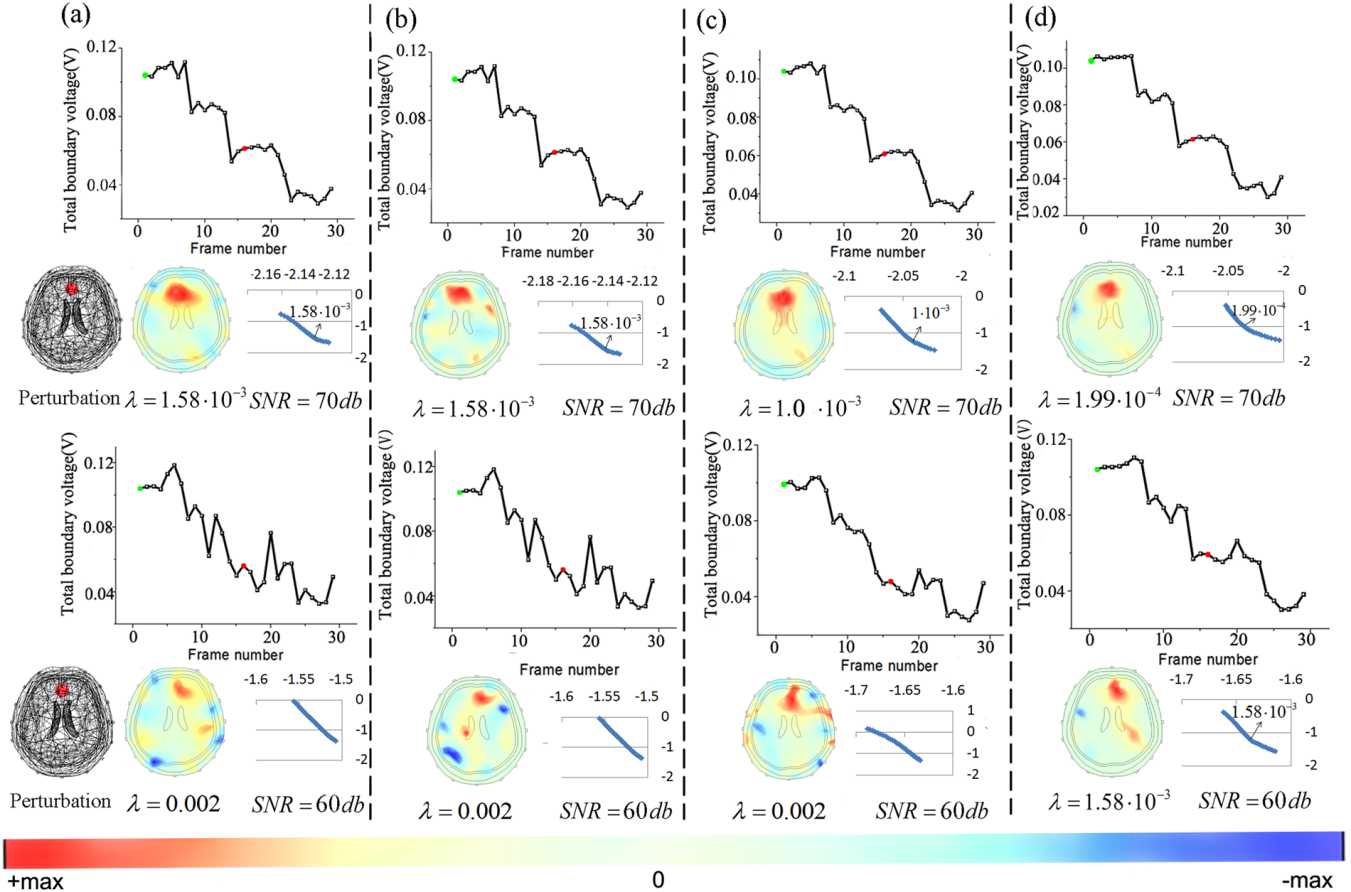
Simulation results of different reconstruction methods with Gaussians noise: the total boundary voltage, EIT images and L-curve obtained by GR (**a**), PR (**b**), SR (**c**) and the proposed SPR (**d**).

Figure 3 shows the boundary voltage changes and EIT images obtained by different methods. Figure 3(a) shows the results of GR and it can be seen EIT signals distort after we add Gaussian noise. When the SNR decreased to 60db, large artifacts which affect the identification of the perturbation appear in the images. In addition, the L-curve method fails to provide an optimal regularization parameter. So we use the hieratical method for the second best. Several values were tried and we finally set λ at 0.002. Although this unsystematic method is simple, it will deteriorate the reconstruction accuracy, becoming another reason that worsens the image quality besides Gaussian noise. For PR, Fig. 3(b) shows it improved image quality by suppressing the artifacts in the skull and scalp domain. And from Fig. 3(c), we know SR also yielded better results compared with GR. Random oscillations in EIT data sets were suppressed and the image artifacts and shape error were reduced. However, neither PR nor SR could correctly reconstruct the intracranial perturbation when the SNR of the measurements was 60db. For the results of SPR (see Fig. 3(d)), with boundary measurements denoised for twice, the L-curve method worked again. And the refined total boundary voltage curve and reconstructed image entitled us to identify the cerebral pathological change.

Based on the reconstructed images, we utilized the qualitative indicator identifiability and three quantitative metrics including image artifacts (IA), location error (LE) and shape error (SE) to evaluate the image quality. To show the metrics with the format of ‘mean ± SD’, we selected another two points near the current frame to reconstruct EIT images and repeated the evaluation process. The results are shown in Table 1. For the identifiability, we presented the images to 5 EIT researchers who were not involved in this work and asked them whether there was an intracranial perturbation in the image. When the Gaussian noise is low (70db), they all thought EIT images reconstructed by different methods were in high quality and the identifiability was good. But when the SNR decreased to 60db, identifiability of GR, PR and SR became bad. For SPR, due to its improved robustness, it recovered the lesion so its identifiability was improved. For quantitative evaluation results, when the SNR is 70db, SPR demonstrated minimal errors in IA (0.313 ± 0.01, n = 3), LE (0.009 ± 0.002, n = 3) and SE (0.322 ± 0.01, n = 3) compared with other methods. And if we define the sum of IA, LE and SE as the image error and use the results of GR as the reference, it can be calculated that PR, SR and SPR respectively reduce image error by 2.9%, 16.9% and 25.6%. When the SNR is 60db, EIT images of GR, PR and SR are so noisy that we can’t correctly calculate the IA, LE and SE. However, SPR can recover the perturbation and the IA, LE and SE in this case are 0.423 ± 0.06, 0.058 ± 0.003 and 0.313 ± 0.01.

**Table 1:** Image evaluation results of different reconstruction methods. Corresponding images are shown in Fig. 3.

Methods	Identifiability	Image Artifacts	Location Error	Shape Error	Image Error
GR(70db)	Good	0.396 ± 0.02	0.012 ± 0.002	0.478 ± 0.01	0.886
PR(70db)	Good	0.370 ± 0.01	0.012 ± 0.002	0.478 ± 0.01	0.860
SR(70db)	Good	0.354 ± 0.01	0.009 ± 0.001	0.373 ± 0.01	0.736
SPR(70db)	Good	0.313 ± 0.01	0.009 ± 0.002	0.322 ± 0.01	0.639
GR(60db)	Bad	—	—	—	—
PR(60db)	Bad	—	—	—	—
SR(60db)	Bad	—	—	—	—
SPR(60db)	OK	0.423 ± 0.06	0.058 ± 0.003	0.313 ± 0.01	0.794

When the SNR is 60db, EIT images of GR, PR and SR are so noisy that we can’t correctly calculate the IA, LE and SE.

### Simulation results with bad contact near the electrode

**Figure 4:**
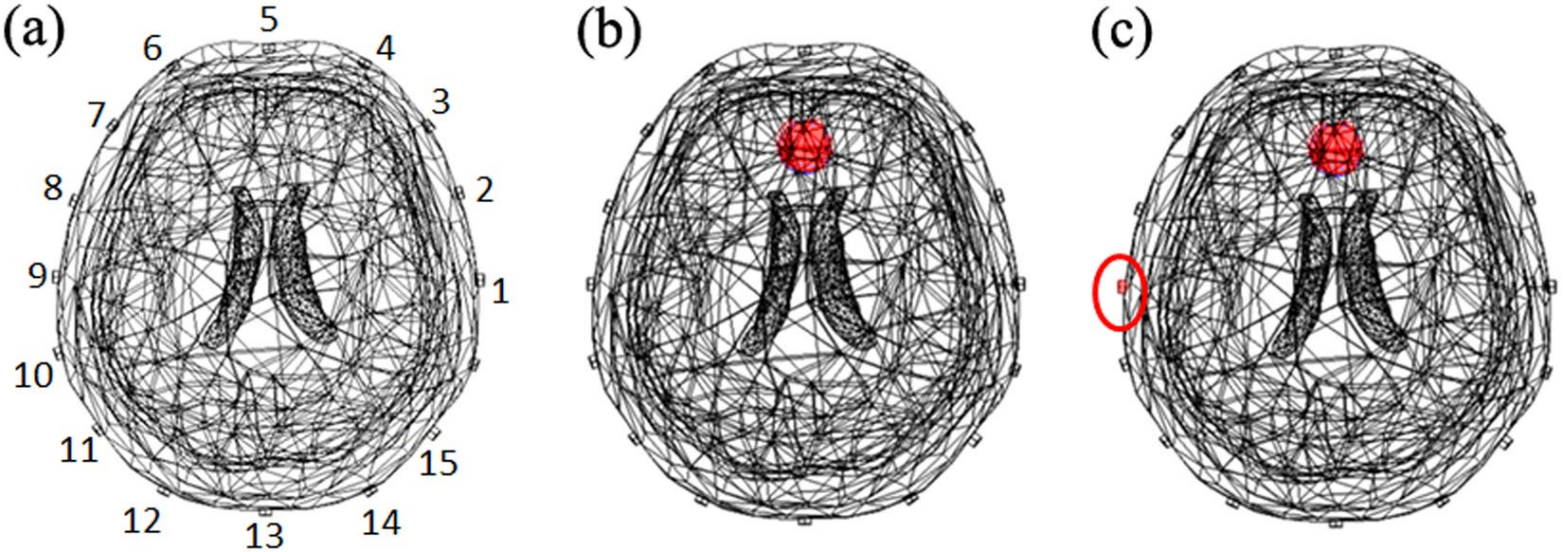
Simulated scenarios of monitoring cerebral hemorrhage with bad contact: Background (**a**), 2 ml intracranial hemorrhage (**b**), 2 ml intracranial hemorrhage with bad contact in electrode 9 (**c**).

**Figure 5:**
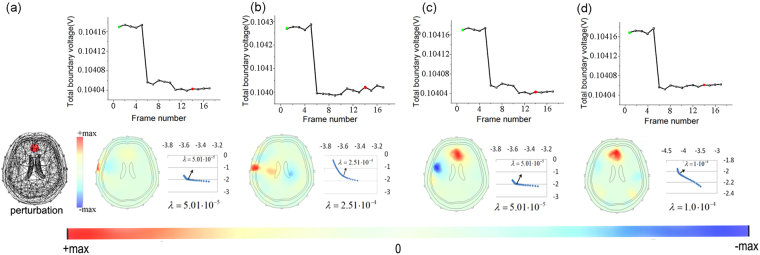
Simulation results of different reconstruction methods at the present of bad contact: the total boundary voltages and reconstructed EIT images obtained by GR (**a**), PR (**b**), SR (**c**) and the proposed SPR (**d**).

In brain injury monitoring, although most patients remain unconscious, the quality of electrodes’ contact with the body may change overtime. Many factors like sweat, patient movement and volatilization of gel can cause conductivity changes near the electrodes, altering the current flowed into the brain^19^. Concerning that EIT is more sensitive to the conductivity changes near the sensing electrodes, the layer packed intracranial perturbations may be at risk of being covered^20^. To simulate this interference, we increased the conductivity value of electrode 9 and the simulations were demonstrated in Fig. 4. Figure 5 shows the resulting total boundary voltages and reconstructed images by different methods.

From Fig. 5 (a), we can see the EIT image reconstructed by GR can’t show the wanted pathological change. As expected, the intracranial perturbations were covered by the conductivity changes near the electrodes. For PR, it recovered the lesion but the EIT image had a lot of artifacts. And the reason may be that PR utilized the optimal regularization parameter chosen for reconstructing the interferences. For SR, although PCA is employed to denoise EIT data, it can’t reconstruct the correct image (see Fig. 5(c)). And it may be blamed on the fact that signal processing methods like PCA didn’t consider the priori of the subspace distribution of the interference. From Fig. 5(d), we know compared to the above methods, SPR got more reliable and accurate EIT images.

### *In-vivo* experiments

We used EIT to monitor the twist-drill drainage operation for patients with acute or chronic subdural hematoma. The experiments were approved by the Research Ethics Committee of the Fourth Military Medical University (FMMU-E-III-001(1–7)) and registered at Medresman.org (No. ChiCTR-DDD-16008272). We confirmed all experiments were performed in accordance with relevant regulations and informed consent had been obtained from all participants. In the experiments, influx and efflux of irrigating fluid (5% dextrose in water, D5W) was performed to evacuate the hematoma around subdural region and simultaneously we used brain EIT to detect intracranial conductivity changes.

**Figure 6:**
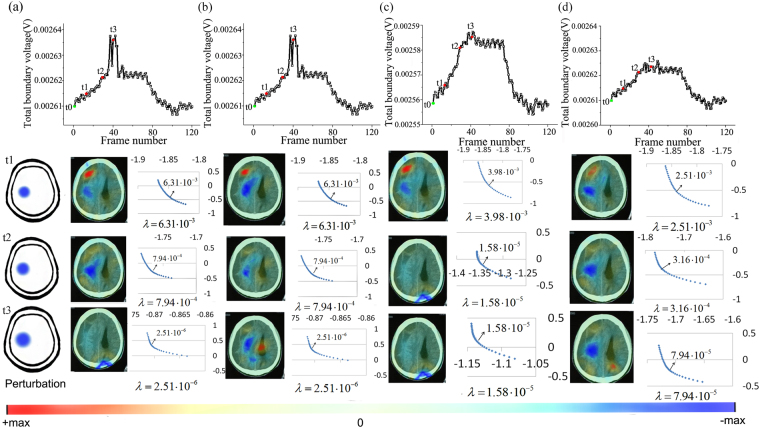
Brain EIT monitoring results of different methods in the twist-drill drainage operation: the total boundary voltages and reconstructed EIT images obtained by GR (**a**), PR (**b**), SR (**c**) and the proposed SPR (**d**).

Figure 6 shows the monitoring results of different methods. The results of GR are shown in Fig. 6(a). We can see the total boundary voltage increased in the influx phase and decreased in the efflux phase, well depicting the cycle of the irrigation and drainage procedure. But a part of the signal becomes abnormal in the plateau. For the reconstruction, we set the reference at t0 when no intervention was performed and selected three points (t1, t2 and t3) in the influx phase as the current frames. For GR, at t1, the amount of inflowed DW5 was so small that only a slight brain conductivity change was induced. So with low SNR, reconstruction of GR at this point was prone to image artifacts. At t2, the EIT measurements and reconstructed images were in good quality. At t3, patient probably moved his head and the electrodes’ pressure on the scalp were changed and it could affect the contact impedance, resulting in incorrect measurements^21^. In this case, GR can only reconstruct the interferences near the electrodes while the intracranial conductivity changes covered. For PR, we can see from Fig. 6(b) that compared with GR, it improved the image quality by eliminating artifacts in skull and scalp domain. But it failed to reduce the reconstruction error caused by improper regularization parameter. For the results of SR, they are shown in Fig. 6(c). With the signal processing method, SR improved reconstruction by suppressing random noise at t1. But it can’t recover the correct perturbation at t3 either. And to our surprise, the PCA method caused wrong reconstruction at t2. From Fig. 6(d), we can see among the three methods, SPR demonstrated best performances. Stable measurements and accurate reconstruction images were obtained after the process and the problem caused by bad contact of electrode was settled with the reconstruction priori information.

## Discussion

To optimize the results of EIT in the application of brain injury monitoring, we proposed a novel reconstruction process SPR. In SPR, we applied the algorithm priori information to help de-noise EIT data and used signal processing for modifying algorithm parameter selection. The experimental results and significance of SPR are summarized as follows.

From the experiments, we know both signal processing methods and algorithm priori information can improve brain EIT image quality. In the simulation experiments, the application of the two techniques respectively reduced image error by 2.9% and 16.9%. For SPR, it reduced image error by 25.6%, which means in SPR, signal processing and algorithm priori information were combined to produce synergic improvements on continuous imaging of brain EIT. As for the significance of SPR, when the level of Gaussian noise is not very high, it can reduce artifacts and shape error. And as the SNR of the signal decreased to 60db, SPR contributes to the identity of intracranial perturbations. The processed total boundary curve has a more evident decrease trend and can work together with the refined EIT image to help us interpret the results. For the bad contact problems, SPR can efficiently recover the image as long as the interferences can be differentiated by the structural priori information. Although this method may become invalid as the signals become noisier, its significance lies in enhancing the robustness of brain EIT reconstruction. And we hope as the optimization of electrode, hardware and algorithm accrued, brain EIT can become stable in the clinical application.

There are several technical problems in our work. First, this paper is limited to provide an optimized reconstruction process. We selected PCA from several signal processing methods based on our previous work^22^ but more advanced signal processing methods should be further investigated. In addition, we only test two typical errors in the experiments. Other interferences like electrode movement are not analyzed. In the future, a more systematic study on the character of interferences in brain EIT monitoring should be conducted and we wish to find the corresponding compensation solutions. Then we can incorporate them into SPR and make the reconstruction method more powerful.

Third, we used L-curve method to choose regularization parameter, which could decrease the imaging speed. In the simulation experiments, it took us 3.03 ± 0.02 s to reconstruct an image (The inverse model has 982 elements and the computer has 32GB RAM and 4.2 GHz CPU). As the development of brain injury will not be very fast, the temporal performance of SPR is acceptable.

## Methods

### Phantom and EIT hardware

In simulation experiments, a realistic 3D head model incorporated with scalp, skull, CSF, parenchyma and ventricle was utilized^23–25^. It has 28000 elements and 16 electrodes evenly fixed on its surface. To reduce the modeling error, structure-based conductivity distribution was applied to solve the forward problem. For human experiments, we employed the EIT system developed by our group to monitor the twist-drill drainage operation. Its measuring accuracy is 0.01% and the common mode rejection ratio of this system is over 80db^26^. For more detailed surgery and measurement process, we can refer to Dai^6^. He reported results of several patients and we choose one case that contains a typical interference of bad electrode connection to test our method.

### The signal preprocessing method PCA

Principal component analysis method performs a mathematical process to transform many correlated variables into fewer unrelated variables. The unrelated variables are called principal components, of which the lower-order principal component contains the most important information of the data. So based on PCA, we can suppress the noise by eliminating the higher-order principal elements. In PCA, we seek the linear combination of elements of EIT voltage data $U$ with the maximum variance. That is to say, a weighting matrix $v$ should be found that satisfies:

1 $$\max \,E(||{U}^{T}v-E({U}^{T}v)|{|}^{2})$$

and (4) can be written as:

2 $$E(||{U}^{T}v-E({U}^{T}v)|{|}^{2})={v}^{T}E((U-E(v)){(U-E(v))}^{T})v={v}^{T}Rv$$

where $R$ is the covariance of $U$, and $v$ is of unit length with ${v}^{T}v=1$.

### Framework of SPR

**Figure 7:**
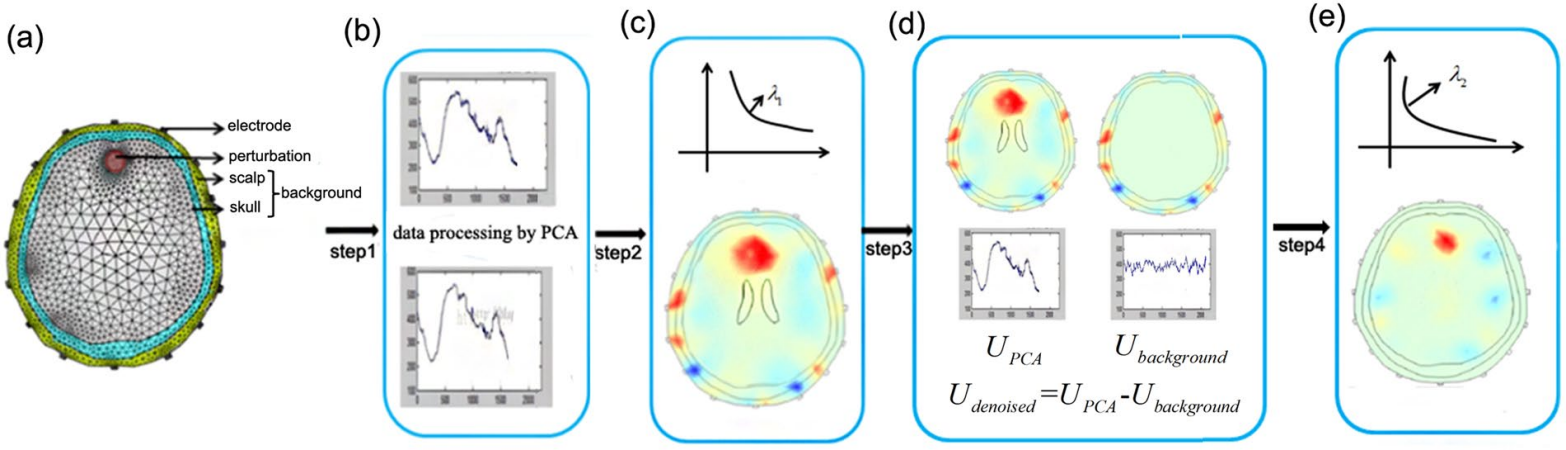
Framework of SPR: (**a**) the predefined perturbation, (**b**–**e**) step1-step4 of SPR.

As displayed in Fig. 7, the frame work of SPR has four steps.

- Step1: PCA is performed to suppress random noise
  3 $${U}_{PCA}={F}_{PCA}(U)$$
  where ${F}_{PCA}$ represents the PCA operator and ${U}_{PCA}$ is the processed EIT data.
- Step2: Reconstruct an initial image
  4 $${\sigma }_{initial}={({J}^{T}J+{\lambda }_{1})}^{-1}{J}^{T}{U}_{PCA}$$
  where ${\sigma }_{initial}$ represents the initial reconstruction result, $J$ is the Jacobian matrix and ${\lambda }_{1}$ is the regularization parameter chosen based on ${U}_{PCA}$.
- Step3: Eliminate the boundary voltage changes of the background (skull and scalp domain) ${U}_{background}$ from EIT measurements
  5 $${U}_{denoised}={U}_{PCA}-{U}_{background}={U}_{PCA}-J{\sigma }_{background}$$
  where ${U}_{denoised}$ represents the final denoised data and ${U}_{background}$ is the conductivity changes in background of the ${\sigma }_{initial}$.
- Step 4: Re-select a regularization parameter and accomplish the final reconstruction

6 $${\sigma }_{SPR}={({J}^{T}J+{\lambda }_{2})}^{-1}{J}^{T}{U}_{denoised}$$

where ${\sigma }_{SPR}$ represents the reconstruction results of SPR and ${\lambda }_{2}$ is the local optimal regularization parameter selected based on ${U}_{denoised}$.

### Regularization parameter selection methods

For the regularization parameter selection methods, the systematic method L-curve is widely implemented^27^. Its principle is to find a tradeoff between the data mismatch and the regularization penalty. When the measurements become very noisy, systematic methods for regularization parameter selection like L-curve are not applicable. In this case, we choose the λ using the heuristic method^28^. Considering the measurements are quite noisy, we make the test scope within the range 1e-4 to 1e-1. 30 values are tested in each reconstruction and we set the λ with the value that can produce the best reconstruction results.

### Evaluation metrics for EIT image

In the experiments, three quantitative evaluation metrics including image artifacts, location error and shape error are used to evaluate EIT image quality^29^. To introduce the metrics, we first make some definitions. The abbreviation PP represents the projection area of the predefined perturbation and RP is the reconstructed perturbation. RP contains elements in the EIT image whose amplitudes of conductivity changes exceed 50% of the peak value of the reconstructed conductivity changes.

Image artifacts (IA): The IA is the ratio between the regions outside RP and the whole image:

7 $$IA=\frac{mean|{\sigma }_{UR}|}{mean|{\sigma }_{A}|}$$

where $mean|{\sigma }_{UR}|$ represents the mean of the conductivity changes of elements outside RP, and $mean|{\sigma }_{A}|$ is the mean of the conductivity changes of elements in whole reconstructed image.

Location error (LE): The LE is the positional mismatch between reconstructed and predefined perturbations.

8 $$LE=\frac{|{d}_{RP}|}{|{R}_{PP}|}$$

where ${d}_{RP}$ represents the position shifts between the gravitational center of the RP and that of PP. And ${R}_{PP}$ denotes the radius of PP.

Shape error (SE) is calculated as:

9 $$SE=\frac{|{A}_{RP}-{A}_{PP}|}{|{A}_{RP}|}$$

where ${A}_{RP}$ denotes the area of the reconstructed perturbation and ${A}_{PP}$ is the area of the predefined perturbation.
